# Depressive symptoms in older adults: the role of physical activity and social support

**DOI:** 10.47626/2237-6089-2020-0093

**Published:** 2021-10-22

**Authors:** Carlos Alberto Moreno Moya, Fernanda Cunha Soares, Rodrigo Antunes Lima, Mauro Virgílio Gomes de Barros, Jorge Bezerra

**Affiliations:** 1 Escola Superior de Educação Física Grupo de Pesquisa em Estilo de Vida e Saúde Universidade de Pernambuco Recife PE Brazil Escola Superior de Educação Física , Grupo de Pesquisa em Estilo de Vida e Saúde (GPES), Universidade de Pernambuco , Recife , PE , Brazil .; 2 Institute of Sport Science University of Graz Graz Austria Institute of Sport Science , University of Graz , Graz , Austria .

**Keywords:** Depression, exercise, psychogeriatrics, social support

## Abstract

**Objective:**

To evaluate the role of social support in the association between physical activity (PA) in its various domains and depressive symptoms in older adults.

**Methods:**

This was a cross-sectional study involving 399 older adults seen at basic health units in the municipality of Recife, state of Pernambuco, northeastern Brazil. Linear regression was implemented using the forward method to assess the association between PA and its domains and depressive symptoms, as well as to test the possible moderating component of social support in this association.

**Results:**

Of the total sample, 17.6% reported not practicing PA; 6.7% presented depressive symptoms, with a mean score of 1.59 points. A total of 18.6% of the older adults assessed reported not having social support. Older adults who did not practice total, transportation or domestic PA showed increased scores of depressive symptoms when compared to older adults who practiced PA in these domains. Older adults with social support also had fewer depressive symptoms. Practicing domestic, leisure and total PA, and/or having social support, decreased depressive symptoms in older adults. Having social support or practicing PA decreased depressive symptoms in older adults in a similar way.

**Conclusions:**

Older adults with social support and who practiced PA, especially domestic and total PA, presented the lowest scores of depressive symptoms. The data showed the importance of providing elements to strengthen social support and PA practice in older adults living in poor communities as a strategy to prevent and reduce depressive symptoms.

## Introduction

Population aging has been growing at an accelerated rate in recent decades, due to changes in demographic structures, low fertility, and increased life expectancy. According to the World Health Organization (WHO), the world population over 60 years of age will double from 11 to 22% between 2000 and 2050. It is estimated that by 2020 there will be 200 million people over the age of 60 in Latin America, and that number should rise to 310 million in 2050. ^1^ By 2025, Brazil will be the sixth country with the highest number of older adults in the world, ^2^ and in the year 2060, the state of Pernambuco will have a population of 3,168,161 older adults, accounting for 31.86% of the northeastern population. ^3^

Even though aging is not synonymous with illness, there is an evident increase in the occurrence of chronic non-communicable diseases among older adults, even with the emergence of different public health policies that encourage healthy aging strategies. ^4^ The WHO estimates that 47 million of older adults have dementia or cognitive impairment, of which 60% live in developing countries such as Brazil. ^5^

Depressive disorders are mood disorders that cause a persistent feeling of sadness and loss of interest. ^6^ As important as seeking treatment for depression in older adults is to identify the factors that precede the chronic phase of the disease. ^7^ Depression tracking carried out through surveying depressive symptoms has been widely used in epidemiological studies. ^8^ The prevalence rates of this condition vary from 20-56% in outpatients, through 23-42% in hospitalized older adults, to 11-52.6% in community older adults. ^9^

Physical activity (PA) seems to be an effective tool in treating and preventing depression and its symptoms. ^10^ PA programs for older adults substantially reduce the severity of depressive symptoms, ^11^ tend to be well tolerated, ^12^ and increase physical fitness, in addition to being among the treatment options preferred by patients. ^13^ Conversely, it is known that physical inactivity in older adults leads to loss of functionality, autonomy and independence, changes in behavior and personality, in addition to social and occupational impairment, thereby leading to the emergence of pathologies such as Alzheimer’s disease, depression and dementia, among others. ^14^

Studies suggest that the effect of PA on depression symptoms occurs through three mechanisms: neurobiological, psychological and social. ^15^ In the neurobiological mechanism, PA seems to increase brain-derived neurotrophic factor (BDNF), noradrenaline and serotonin (hormones responsible for well-being), leading to improvement in depression. ^16^ From the psychological point of view, PA is able to improve negative self-assessment, low self-esteem and low self-confidence among older adults. ^17^ Finally, the third mechanism by which PA acts on depression and related symptoms is the social mechanism. However, evidence supporting this mechanism is still not well-stablished. Some authors suggest that PA performed in groups promotes connection between participants, increasing the social support network and thereby improving depressive symptoms. ^18 , 19^ In contrast, other studies reported no differences in depression improvement when comparing exercises performed individually at home vs. exercises performed in groups in sports centers. ^20^

Understanding the relationship between PA and depressive symptoms in older adults, as well as the role of social support in this association, and taking into account cultural and regional differences can contribute to planning care policies aimed at healthy aging, therefore helping in the treatment and prevention of depression in older adults. Thus, the objective of the present study was to assess the role of social support in the association between PA and depressive symptoms in older adults seen at basic health units in the municipality of Recife, state of Pernambuco, northeastern Brazil.

## Methods

This was a cross-sectional study, part of a broader research project titled “EUGERON - Effectiveness of interventions for health promotion in frail older adults with chronic non-communicable diseases in primary healthcare in Recife: A randomized community trial.” ^21^ The project was approved by the human research ethics committee at Universidade de Pernambuco (opinion no. 1,722,850). All research participants signed an informed consent form before their enrollment in the study.

A population-based survey was carried out through household sampling to perform the study, resulting in a sample that was representative of the older adult population residing in the coverage area of the Family Health Strategy in the city of Recife, state of Pernambuco, northeastern Brazil. The target population was limited to older adults aged 60 or over, living in the coverage area of the Family Health Strategy in the city of Recife.

The sample size was established considering the interest in estimating the prevalence of several health variables; therefore, the expected prevalence was established at 50%. In addition, the following parameters were defined: error of 4 percentage points, 95% confidence intervals (95%CI), and 80% power. Considering these factors, sample size calculation resulted in a minimum number of 597 participants using a sample design effect (deff = 1.5). Then, it was decided that the minimum sample should be inflated by an additional 30% to ensure sufficient statistical power for the analyses and to deal with possible refusals. Respecting these parameters, the sample size to be reached was established at 770 participants.

Participant selection was carried out by sampling in two stages, with basic health units being drawn in the first stage and households in which the older adults resided in the second stage. In the first stage, 21 basic health units (primary sample units) were selected from the 130 existing units in Recife. In the second stage, the homes where the older adults resided (sampling unit in the second stage) were identified in the territory by the research team in cooperation with the health team of the basic health unit itself.

Data were collected between the months of October and November 2017. A total of 399 older adults were monitored due to unforeseen events which occurred during the collection. Therefore, *a posteriori* sample calculation was carried out for this study, and it was possible to detect a significant risk ratio greater than 1.5, with 95%CI and statistical power greater than 80%.

A questionnaire for use in face-to-face interviews was built specifically for use in this project. It included questions already used in other instruments standardized and validated for use with Brazilian older adults. The instrument was applied by evaluators previously trained by the project researchers. Through this questionnaire, we obtained information on sex (male, female), age, maternal education level (≤ 8 years of education, > 8 years of education), family income (none, < 2 minimum wages, ≥ 2 minimum wages), marital status (married/with partner, no partner), skin color (white, black, other) and self-perceived health (good, bad).

The outcome of the present study was depressive symptoms. The presence of these symptoms was assessed using the five-item Geriatric Depression Scale (GDS-5), originally validated by Rinaldi et al. ^22^ and later by Almeida & Almeida ^23^ for the older adult population of Recife. Depressive symptoms were presented as continuous variables for the main analyses, with a score ranging from 0 to 5 points. A cut-off point of ≥ 4 points was used to determine the presence of depressive symptoms.

Support social was evaluated by a specific domain of the Tilburg Frailty Indicator (TFI) questionnaire, used to assess frailty ^24^ and validated for the Brazilian population. This domain consists of three questions: a) Do you live alone? (yes/no); b) Do you have enough support from others? (yes/no); c) Do you miss having people by your side? (yes/sometimes/no). The following criteria were used to calculate a score: answers “no” were assigned a value of 0; a value of 1 was assigned to answers “yes” to the first two questions and to answers “sometimes” in the third question; finally, a value of 2 was assigned answers “yes” to the third question. The score could vary from 0 to 4 points. Older adults without social support were considered to be those who obtained a score > 2 points.

The International Physical Activity Questionnaires (IPAQ), validated for the Brazilian population, ^25^ were applied to assess the time spent on PA in the different domains: transportation, domestic, and leisure time. PA was categorized considering as inactive older adults those who reported zero minutes of PA in each respective domain. ^26^ Total PA was also assessed in the same way: older adults who scored zero for leisure, transportation or domestic PA were characterized as inactive.

The Stata statistical package (version 14.0) for Windows was used for data analysis. A significance level of p ≤ 0.05 was adopted in all inferential analyses. Multiple imputation using the Multivariate Imputation by Chained Equations (MICE) package was used to impute missing data due to the lack of some information about the older adults. Missing values were imputed 5 times each to improve accuracy, resulting in 5 imputed data sets.

**Figure 2:**
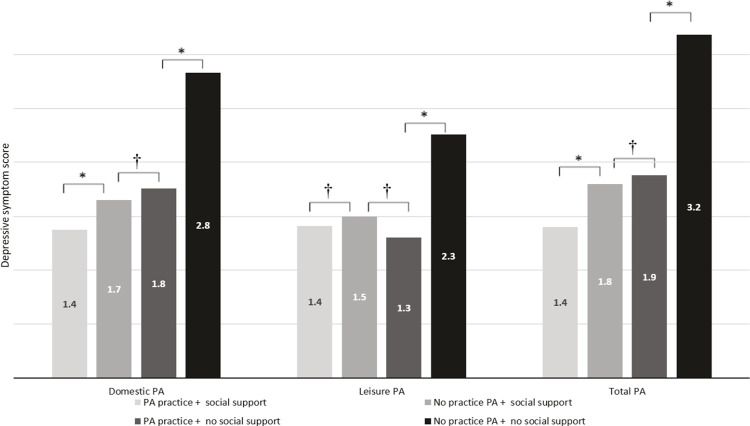
Mean scores of depressive symptoms according to practice of PA and social support in older adults residing in the Family Health Strategy coverage area, Recife, state of Pernambuco, Brazil. Analyses adjusted for gender, age, self-perceived health. PA = physical activity. * p < 0.05; † p ≥ 0.05.

After performing multiple imputation, frequency distribution, measures of central tendency (means/medians) and dispersion (standard deviation/interquartile range [IQR]) descriptive analyses were performed. The histogram of depressive symptoms data shows a normal distribution of the data. The independent *t* -test was used to assess differences in means. Linear regression with the forward method was used to assess the association between PA and its domains and depressive symptoms (results presented further below, in Table 3 ). We tested the interaction between social support (having social support or not having social support) and PA practice (yes and no) in different domains (domestic, leisure and transportation) independently. When interaction was observed, we estimated the mean predictive value of the depressive symptom score for each category of interaction using the margins function available in Stata version 14 after conducting the linear regression (results presented further below, in Figure 2 ).

**Table 3:** Multivariate linear regression: association between physical activity domains and depressive symptoms in older adults residing in the Family Health Strategy coverage area, Recife, state of Pernambuco, Brazil

Variable/category	Crude model	Adjusted model I	Adjusted model II	Adjusted model III	Adjusted model IV
Social support					
Yes	1	1	1	1	1
No	**0.65 (0.35-0.94)**	**0.64 (0.34-0.93)**	**0.63 (0.33-2.45)**	**0.57 (0.28-0.87)**	**0.63 (0.33-0.91)**
Transportation PA					
Yes	1	1	-	-	-
No	0.22 (-0.02-0.45)	**0.25 (0.01-0.49)**	-	-	-
Domestic PA					
Yes	1	-	1	-	-
No	**0.35 (0.11-0.59)**	-	**0.42 (0.17-0.66)**	-	-
Leisure PA					
Yes	1	-	-	1	-
No	0.26 (-0.01-0.53)	-	-	0.23 (-0.03-0.49)	-
Total PA					
Yes	1	-	-	-	1
No	**0.44 (0.14-0.73)**	-	-	-	**0.54 (0.24-0.83)**

Values presented as β (95% confidence intervals). Model I: transportation PA + social support + adjustments (gender, age, self-perceived health); Model II: domestic PA + social support + adjustments (gender, age, self-perceived health); Model III: leisure PA + social support + adjustments (gender, age, self-perceived health); Model IV: total PA + social support + adjustments (gender, age, self-perceived health).

## Results

A total of 397 older adults (77.3% women) living in the Family Health Strategy coverage areas in the city of Recife participated in the present study, with a mean age of 72.8±8.1 years. Most of the older adults had low education (90.4%) and received less than two minimum monthly salaries (87.4%). Also, the majority of the participants (59.1%) did not live with a partner, and 74.4% had a poor self-perception of their health ( Table 1 ).

**Table 1:** Characteristics of the older adults residing in the Family Health Strategy coverage area, Recife, state of Pernambuco, Brazil (n = 397)

Variable/category	n	%
Sex		
Male	90	22.7
Female	307	77.3
Education		
≤ 8 years	358	90.4
> 8 years	38	9.6
Income		
None	29	13.8
< 2 minimum salaries	293	73.6
≥ 2 minimum salaries	50	12.6
Marital status		
Married/with partner	163	40.9
No partner	235	59.1
Skin color		
White	118	29.6
Black	103	25.9
Other	177	54.6
Self-perceived health		
Good	102	25.6
Bad	296	74.4

The median time spent on transportation PA was 6.7 minutes per day (IQR: 0.0-25.7), on domestic PA 15.0 minutes per day (IQR: 0.0-80.0), and on leisure time PA was 0.0 minutes per day (IQR: 0.0-4.4).

It was found that 18.6% (95%CI 14.9-22.8) of the older adults did not have social support. When analyzing the factors that comprised social support separately, the following results were obtained: 17.0% (95%CI 13.5-21.1) lived alone; 46.4% (95%CI 41.4-51.4) reported missing having people by their side; and 17.2% (95%CI 13.7-21.4) did not have enough support from other people.

The prevalence of older adults who did not practice PA was 17.6% (95%CI 14.1-21.6). When assessing the prevalence in the different domains of PA, it was observed that 34.2% (95%CI 29.5-39.0) of the older adults did not practice domestic PA; 40.7% (95%CI 36.0-45.6) did not practice transportation PA; and 73.1% (95%CI 68.5-77.2) did not practice PA during leisure time.

**Figure 1:**
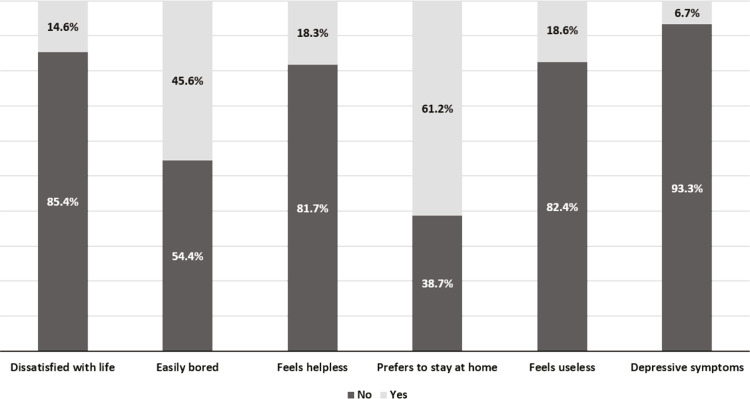
Prevalence of the presence of depressive symptoms and their domains in older adults residing in the Family Health Strategy coverage area, Recife, state of Pernambuco, Brazil.

A mean score of 1.59 points was obtained for depressive symptoms in the older adults assessed, with a range between 0 and 5. The percentage of older adults who were dissatisfied with life was 14.6% (95%CI 11.3-27.7), while 18.3% (95%CI 14.7-22.5) reported feeling helpless, 18.6% (95%CI 15.0-22.9) felt useless and 45.6% (95%CI 40.6-50.7) said they were easily bored. More than half of the older adults (61.2%, 95%CI 56.2-66.1) preferred to stay at home rather than go anywhere else. When evaluating older adults who answered “yes” to 4 or more of the previous questions, it was found that 6.7% (95%CI 4.5-9.7) of the older adults had depressive symptoms ( Figure 1 ).

Older adults who had a poor self-perceived health presented a higher mean of depressive symptoms than older adults who had a good perception of their health. Likewise, older adults without social support had a higher mean of depressive symptoms than older adults with social support. Older adults who did not perform domestic or total PA also had more depressive symptoms than older adults who practiced PA ( Table 2 ).

**Table 2:** Differences in means of depressive symptoms according to sociodemographic factors, social support and physical activity domains in older adults residing in the Family Health Strategy coverage area, Recife, state of Pernambuco, Brazil

Variable/category	Depressive symptoms		p-value*
	Mean	SD	
Sex			
Male	1.4	1.0	0.182
Female	1.6	1.2	
Education			
≤ 8 years	1.6	1.2	0.954
> 8 years	1.6	1.2	
Marital status			
Married/with partner	1.7	1.1	0.350
No partner	1.5	1.2	
Self-perceived health			
Good	1.1	1.0	**< 0.001**
Bad	1.7	1.2	
Social support			
Yes	1.5	1.1	**< 0.001**
No	2.1	1.4	
Transportation PA			
Yes	1.5	1.1	0.070
No	1.7	1.3	
Domestic PA			
Yes	1.5	1.1	**0.005**
No	1.8	1.3	
Leisure PA			
Yes	1.4	1.0	0.051
No	1.7	1.2	
Total PA			
Yes	1.5	1.1	**0.004**
No	1.9	1.4	

* p-value estimated by the independent *t* -test.

Older adults who did not practice transportation PA showed an increase of 0.25 points (95%CI 0.01-0.49) in the depressive symptom score when compared to older adults who engaged in transportation PA. There was an increase of 0.42 (95%CI 0.17-0.66) in the depressive symptom score in older adults who did not practice domestic PA compared to those who did. When assessing total PA, it was observed that older adults who did not practice any PA showed an increase of 0.54 (95%CI 0.24-0.83) points in the depressive symptom score when compared to older adults who practiced some PA ( Table 3 ).

We also tested an interaction between social support and the different PA domains. There was no interaction between transportation PA and social support (p = 0.945); conversely, interactions between social support and total PA as well as between social support and domestic and leisure PA were observed. Figure 2 presents the depressive symptom scores obtained according to social support and practice of total, domestic, and leisure PA. Regardless of the type of PA assessed, older adults without social support and who did not practice PA showed the highest depressive symptom scores. In other words, engaging in any PA and having social support decreased the depressive symptoms of the evaluated older adults. In the context of domestic and total PA, older adults who practiced PA and had social support presented the lowest depressive symptom scores compared to any other PA domain and social support ( Figure 2 ).

## Discussion

The present study showed that older adults who did not practice PA had higher depressive symptom scores when compared to those who practiced PA. In general, older adults without social support and who did not practice PA presented the highest depressive symptom scores, while practicing PA and/or having social support were associated with the lowest depressive symptom scores in this older adult group. Thus, practicing PA and/or having social support were associated with lower depressive symptom scores in older adults.

The association between PA and its domains and depressive symptoms in older adults here reported supports the findings of a previous meta-analysis ^27^ that suggested an inverse relationship between PA levels and morbidity and mortality from all causes, with stronger associations for domestic PA and leisure PA. In a study conducted in southern Brazil, physically active older adults were 68% less likely to experience depressive symptoms when compared to those who were insufficiently active. ^28^ Evidence suggests that exercise can relieve depression through common neuromolecular mechanisms, including increased expression of neurotrophic factors (BDNF), increased availability of serotonin and norepinephrine, regulation of hypothalamic-pituitary-adrenal (HPA) axis activity and reduction of systemic inflammatory signaling. ^15^ The beneficial effect of PA in older adults can be manifested in improving mood, reducing physiological responses to stress, positive effects on body image, cognitive functioning and self-esteem, in addition to improving sleep quality and promoting greater satisfaction with life. ^29^

Conversely, low social support was associated with higher depressive symptom scores. Older adults who are unable to adapt to different stress factors such as unfavorable environments, lack of social and family support and scarce social resources tend to have more depressive symptoms. ^30^ In contrast, participation in community or family coexistence groups is a protective effect against depressive symptoms, signaling the importance of the social network in maintaining health and preventing and/or treating depression among older adults. ^31^

Older adults with social support and who practiced PA presented the lowest depressive symptom scores, especially in domestic and total PA. Social support can also be achieved through regular participation of older adults in walking and swimming groups, among other PAs, with an even better impact on depressive symptoms, since group PA is recognized as an opportunity for open social communication, with topics that go beyond sport. ^32^ Thus, social interactions related to group activities can help people deal with depressive symptoms and better overcome challenges. The main characteristics of older adults assessed in the present study were low education and low income, which is in line with other studies. ^33^ Individuals with higher education and higher income tend to seek the use of other services such as private care, given that this portion of the population most often has health insurance. Furthermore, although most older adults did not live alone, most of them reported missing having people at their side – it is known that many older adults experience loneliness or feel lonely at this stage of life, even when living with or in the company of family members. ^34^ The perception of older adults that there are people available to give them support and assistance while facing daily demands reduces their tension, fears and concerns about life in general. ^35^

The practice of PA by the older adults was low in all domains. The environment where the study was carried out seems to have no facilitators for practicing PA, especially in leisure time, where the prevalence of physical inactivity was 73.1%. There are no adequate facilities available, the pavement/sidewalk is precarious, leading to accidents along the way, and the neighborhood is not a safe place in the opinion of the older adults. Accessibility to leisure areas and open public spaces with structures suitable for practicing PA, in addition to safe environments, could increase the chances of practicing PA. ^36 , 37^

Some limitations of this study must be mentioned. The first one is the cross-sectional nature of the study, which does not enable to determine the causality between PA, depressive symptoms and social support. Therefore, longitudinal and intervention studies are needed to confirm the direction of these associations. Also, data on depressive symptoms, social support and mainly PA were obtained by self-reported measures, which may lead to memory bias. However, prior and standardized training was offered to recruited interviewers to minimize this potential source of error, and most of them were used to conducting academic-scientific activities and carrying out fieldwork.

## Conclusion

The present data suggest that practicing PA and/or having social support are associated with lower depressive symptom scores, evidencing the importance of providing elements to strengthen social support and PA practice in older adults who belong to vulnerable communities.
